# The Effect of Encapsulated Propyl Propane Thiosulfonate (PTSO) on Apparent Ileal Digestibility and Productive Performance in Broiler Chickens

**DOI:** 10.3390/ani13061123

**Published:** 2023-03-22

**Authors:** Gonzalo Villar-Patiño, María del Carmen Camacho-Rea, Myrna Elena Olvera-García, Arturo Soria-Soria, Julio César Baltazar-Vázquez, Gabriela Gómez-Verduzco, Lourdes Solano, Guillermo Téllez, Aurora Hilda Ramírez-Pérez

**Affiliations:** 1 Programa de Doctorado en Ciencias de la Salud y de la Producción Animal, Facultad de Medicina Veterinaria y Zootecnia, Universidad Nacional Autónoma de México, Avenida Universidad 3000, Coyoacán, Ciudad de México 04510, Mexico; gvillar@gponutec.com; 2 Grupo Nutec, El Marqués, Querétaro 76246, Mexico; 3 Instituto Nacional de Ciencias Médicas y Nutrición “Salvador Zubirán”, Departamento de Nutrición Animal, Tlalpan, Ciudad de México 14080, Mexico; 4 Specialized Animal Nutrition Research Network, Grupo Nutec, San Juan del Río, Querétaro 76814, Mexico; 5 Facultad de Medicina Veterinaria y Zootecnia, Universidad Nacional Autónoma de México, Avenida Universidad 3000, Coyoacán, Ciudad de México 04510, Mexico; 6 Department of Poultry Science, University of Arkansas, Fayetteville, AR 72701, USA

**Keywords:** broilers, propyl propane thiosulfonate, apparent ileal digestibility, amino acids, energy, phosphorus

## Abstract

**Simple Summary:**

There are currently two main concerns in broiler production. The first concern is the production cost, the key to which is improving feed efficiency; the second relates to the environmental impact. Both are vitally important for business sustainability. Some existing nutritional strategies contribute to improving the use of nutrients to achieve better performance; one of these involves adding plant extract additives, as well as their bioactive compounds, to animal feed. These additives improve digestive processes and nutrient absorption. *Allium* species contain sulfur compounds, such as propyl propane thiosulfonate, which has been studied in broilers and was found to enhance the digestibility of energy, fat, and fiber. Nevertheless, as far as we know, there is no scientific research focused on the digestibility of amino acids and minerals, which play an important role in growth and feed efficiency. Moreover, decreasing the levels of nitrogen and other minerals in feces could reduce the amount of pollutants released into the environment. Our results show that the supplementation of encapsulated propyl propane thiosulfonate in the broiler chicken diet improves the apparent ileal digestibility of amino acids and energy compared to the control diet; a positive trend in the digestibility of phosphorus was also found.

**Abstract:**

This study analyzed the effects of different dietary doses of encapsulated propyl propane thiosulfonate (Pe-PTSO) on the apparent ileal digestibility (AID) of nutrients and productive performance in broilers. A total of 100 one-day-old Cobb 500 were housed in battery cages for 20 days. At 10 days of age, the birds were assigned to one of five diets: negative control (P0), 250 mg/kg of Pe-PTSO (P250), 500 mg/kg of Pe-PTSO (P500), 750 mg/kg of Pe-PTSO (P750), and positive control, nicarbazin–narasin (ION). Titanium dioxide was the external marker, which was added to the diets from day 17 to 20. In the birds fed the P250 diet, there was a significant difference (*p* ≤ 0.05) in the AID values for amino acids and energy compared to those that consumed the P0 diet. Furthermore, the P250 diet significantly increased (*p* ≤ 0.05) the average daily weight gain compared to the P0 diet. No significant differences were observed between treatments in average daily feed intake and feed conversion ratio. In summary, the inclusion of 250 mg of encapsulated PTSO per kg in broiler chickens diet improved the digestibility of amino acids and energy, as well as weight gain.

## 1. Introduction

Food security for the world population, which will grow by approximately 20% in the next 25 years, challenges the ability to produce food more efficiently and sustainably [1,2]. Poultry production is considered to be an environmentally and economically sustainable activity due to the ability of poultry to convert feed in meat [3]. However, the feed is considered the major economic cost of animal production, therefore, the scientific research to improve the utilization of nutrients by the broiler chicken evolve continuously. Poultry diets are formulated based on both digestibility and nutrient absorption, which allows us to know their utilization rate by the bird [4,5]. Increasing nutrient digestibility in the diet has been shown to enhance the productive performance of broiler chickens [5,6]. Different formulation strategies have been adopted to optimize the growth and improve feed efficiency of birds [7], such as fecal digestibility, true digestibility, standardized ileal digestibility, and apparent ileal digestibility (AID) [8]; all of them quantified digested dietary nutrients [9]. Currently, there are different feed additives, such as enzymes, prebiotic, probiotic, ionophores, and recently, the use of phytochemicals, which contributes to enhance nutrient digestion [10,11]. Narasin and nicarbazin, ionophore coccidiostats, are frequently used as feed additives in broiler diets; it has been reported that their supplementation increased crude protein digestibility and affects intestinal microbiota, improving gut health of broiler chickens [12]. Likewise, narasin has also been found to enhance feed efficiency in birds reared on different production systems, including caged systems without coccidia infection [13]. Scientific research about nutrient digestion using natural additives is increasing. Phytochemicals are natural plant compounds produced as secondary metabolites, which differ in chemical structure, biological activity, and plant origin [14,15]. These secondary metabolites are considered natural sources of feed additives, as well as natural growth promoters [16], which are generally recognized as safe (GRAS) [17]. They possess significant biological activities that affect gut health, improve intestinal morphology [18,19], modulate gut microbiota, and enhance the metabolic activity leading to improvements in both digestibility and nutrient utilization [18,19,20]. Phytochemicals are categorized into five main groups: terpenoids, polyphenols, phytosterols, alkaloids, and organosulfur compounds [21].

Propyl propane thiosulfonate (PTSO) is an organosulfur compound belonging to the genus *Allium*, derived from the natural degradation of propiin, which is the *Allium* flavor precursor [22,23]. Unlike other sulfur components from this *Allium* genus, PTSO is chemically stable but poorly soluble in water, hence it is necessary to provide it with a specialized carrier to increase its biological availability and absorption [24]. PTSO has been studied as an additive in animal nutrition showing beneficial results on growth performance [25,26,27,28]. In addition, it has antimicrobial effects, which have been demonstrated in vitro and in vivo against *Enterobacteriaceae,* such as *Escherichia coli* and *Salmonella* spp., as well as *Campylobacter jejuni* in broilers [26,28]. Moreover, Peinado, et al. [27] reported that PTSO could modulate intestinal microbiota composition. Furthermore, different immunomodulatory effects of PTSO in broiler chickens have been reported [25]. However, there is limited research about the effect of PTSO on nutrient digestibility in poultry. In this sense, Peinado, et al. [27], who supplemented a broiler chickens diet with PTSO, found an increase in the digestibility of energy, fat, and acid-detergent and neutral detergent fibers.

To the best of our knowledge, there are no studies about the effect of PTSO on the digestibility of amino acids and phosphorus in broiler chickens.

We hypothesized that the inclusion of an encapsulated product of propyl propane thiosulfonate (Pe-PTSO) will improve the AID of amino acids, energy, and phosphorus, in a corn–soybean meal diet. Therefore, the aim of the present study was to evaluate the effect of different doses of Pe-PTSO on AID of amino acids (arginine (Arg), lysine (Lys), leucine (Leu), threonine (Thr), histidine (His), isoleucine (Ile), valine (Val), and phenylalanine (Phe)), energy, and phosphorus, in a corn–soybean meal diet, as well as their effects on growth performance of broiler chickens.

## 2. Materials and Methods

### 2.1. Animals, Diets, and Experimental Design

A total of 100 one-day-old Cobb 500 broiler chickens were housed in a facility equipped with battery cages (48 cm × 80 cm) and provided with manual feeders and automatic nipple drinkers. The environmental temperature was set to 30 °C during the first week; after that, it was maintained between 26 and 27 °C. Relative humidity was set between 55 and 60%.

From 1 to 20 days of age, the birds were fed a corn–soybean meal basal diet (Table 1) that met or slightly exceeded the nutritional needs of chickens of the Cobb 500 lineage. The pre-experimental period lasted from the first day until 9 days of age. At 10 days of age, the birds were weighed and allocated to 1 of 5 treatments in a completely randomized design, as follows: P0—a negative control (basal diet); P250—basal diet + 250 mg/kg of Pe-PTSO; P500—basal diet + 500 mg/kg of Pe-PTSO; P750—basal diet + 750 mg/kg of Pe-PTSO, and ION—a positive control (basal diet + 50 mg/kg of nicarbazin + 50 mg/kg of narasin).

Each treatment had 5 replicates. The experimental unit was the cage with 4 birds in each; the number of birds and replicates were as those reported in previous studies of ileal and total broiler digestibility [27,29,30]. In addition, access to drinking water and feed was provided ad libitum.

### 2.2. Pe-PTSO Supplementation

The PTSO used in this study was a product (Pe-PTSO) encapsulated into a matrix of dextrin and lecithin, having a concentration of 12 g/kg, as determined by gas chromatography–mass spectrometry (GC–MS). The retention time of the chromatography peak was indicated for the PTSO [31]; the databases of the NIST/EPA/NIH Mass Spectra Library, version 1.7 (Gaithersburg, MD, USA), were used. The analysis was carried out in the laboratory of the Center for Research in Applied Sciences and Advanced Technology of the National Polytechnic Institute (IPN, Querétaro, Mexico).

### 2.3. Growth Performance

The body weight (BW) of the birds was recorded at 10 days of age and at the end of the study (20 days of age), and was used to estimate the average daily weight gain (ADG). Feed intake was recorded to calculate the average daily feed intake (ADFI) and the feed conversion ratio (FCR).

### 2.4. Apparent Ileal Digestibility Study

Titanium dioxide was added to the diets at 5 g TiO_2_/kg, as an indigestible marker for the AID study [32]. The diets with TiO_2_ were offered from day 17 to 20. At the end of the experiment, all birds were humanely killed by cervical dislocation [5,33,34]. The content of the ileum at 2.0 cm from the ileocecal valve was collected in sterile bags and preserved at −70 °C in an ultra-low temperature freezer (Thermofisher Scientific, TSX, Waltham, MA, USA) until the analysis. The ileum content was vacuum dried (FreeZone Triad Benchtop Freeze Dryer, Labconco, Kansas City, MO, USA) and pulverized to a particle size of 0.5 mm.

#### Laboratory Analysis

A titanium dioxide reference curve was developed according to that reported by Short, et al. [32]. It was generated using a UV visible spectrophotometer (Agilent 8453/G1103A, Shanghai, China). The diets and ileal content were previously subjected to acid hydrolysis to quantify the amino acids (Arg, Lys, Leu, Thr, His, Ile, Val, and Phe) by ultra-performance high-resolution liquid chromatography using an ACQUITY UPLC system (Waters H-Class, Milford, MA, USA) equipped with a diode array detector, following the AOAC 994.12 method [35]. The quantification of phosphorus (P) was performed using the photometric methodology suggested by AOAC (965.17) [36], using a spectrophotometer (Agilent 8453/G1103A, Shanghai, China). The energy was quantified using an adiabatic bomb (IKA Model C200 basic, Staufen, Germany) according to the ASTM D2015-66 method [37].

The AID of amino acids, energy, and phosphorus was calculated using the following equation:

AID = [1 − [(TiD × NI)/(ND × TiI)] × 100

where TiD is the concentration of TiO_2_ in the diet; NI is the concentration of the nutrient in the ileal digesta; ND is the concentration of the nutrient in the diet; and TiI is the concentration of TiO_2_ in the ileal digesta [38].

### 2.5. Statistical Analysis

The data were subjected to one-way ANOVA using JMP (SAS Institute, Cary, NC, USA, 2019) [39]. Tukey’s test was used for the post hoc analysis. The significance level was set at *p* ≤ 0.05, and a trend was set among *p* > 0.05 and ≤0.10. The initial body weight (IBW) at 10 days was used as a covariate for ADG, ADFI, and FCR. In addition, to determine whether the effect of the different doses of Pe-PTSO was linear, quadratic, or cubic, a follow-up trend analysis using orthogonal polynomial contrasts was performed for the AID data.

## 3. Results

### 3.1. Apparent Ileal Digestibility Study

Table 2 shows the AID of nutrients in broiler chickens fed diets containing different levels of Pe-PTSO. The digestibility of the basal diet used in this study exceeded 90%, which is in concordance with that value reported by An et al. [23], who also used diets based on corn–soybean meal. The AID of Arg, Lys, Leu, Thr, His, Ile, Val, and Phe, as well as energy, was significantly higher (*p* ≤ 0.01) in birds feed the P250 diet than in the birds fed the P0 diet. Overall, the average increase in the AID of amino acids was 2.3%; the lowest increment was observed in Arg (1.70%), and the greatest increase was observed in Ile (3.04%). In the P250 diet, the energy digestibility was 0.15 Mcal/kg higher than that in the P0 diet. Moreover, the P250 diet resulted in greater digestibility (*p* ≤ 0.05) for Arg, Lys, and Thr, as well as for energy, compared to the P750 diet.

Regarding phosphorus digestibility, there was a trend (*p* = 0.06) to improve it when 250 mg/kg Pe-PTSO was added to the diet. The ION and P500 treatments did not show differences (*p* > 0.05) for any nutrient evaluated.

The polynomial contrasts between the treatments are summarized in Table 3. There was a significant cubic positive response (*p* ≤ 0.05), rather than a linear or quadratic response, for the digestibility of all nutrients analyzed. As we mentioned above, the P250 diet resulted in the highest values for digestibility in all cases.

### 3.2. Growth Performance

The average initial body weight (IBW) of the chickens at 10 days of age is 286.6 gr.

The ADG was higher (*p* ≤ 0.05) in the broilers fed the P250 diet than in those fed the P0 diet, while the P500, P750, and ION groups did not show any differences (*p* > 0.05).

The ADFI showed a similar trend than ADG (*p* = 0.06), the highest value for P250 (74.92 g/d) and the lowest with P0 (67.87 g/d). Nevertheless, this increment against P0 was not observed with P500, P750, or ION birds.

The FCR was not affected (*p* > 0.05) by treatment (Table 4). It should be noted that no bird mortality was observed in the experimental period.

## 4. Discussion

### 4.1. Apparent Ileal Digestibility

Improving nutrient digestibility in the diets of poultry chickens has shown a positive impact on nutrition, productivity, as well as on the environment. Thus, increasing nutrient digestibility not only improves the optimal use of nutrients, but also constitutes a significant component of sustainable animal protein production [1,7]. The use of phytochemicals as additives in animal nutrition enhances digestive enzyme activity and productive performance [40,41].

It has been reported that PTSO improves the digestibility of energy, fat, and acid detergent and neutral detergent fibers in broiler chickens diets [27]. Nevertheless, to our knowledge there is not scientific research regarding the effect of PTSO on the digestibility of other nutrients, such as amino acids and phosphorus.

In this study, we observed that P250 was the only dose that positively affects the AID of amino acids and energy. We do not have enough fundamentals to explain why higher doses of PTSO did not increase the AID of nutrients. We could suggest that the responses to PTSO doses appear to behave under the law of diminishing returns, which states that at higher doses, the increases in the response variable lessens, until it reaches a point in which it begins to decrease [42]. Assuming this statement were true, then it is necessary to explore if there is a dose among P250 and P500 that increase the AID of nutrients. Regarding to ION treatment, our data suggests that it has not significant effect on AID.

The beneficial effect of Pe-PTSO on amino acid digestibility is important because amino acids are critical dietary components regulating physiological, metabolic, and structural functions [43]. In this sense, a study conducted by Brzóska, et al. [44], who fed broiler chickens by adding an extract of *Allium sativum* in the feed, an increase in crude protein content in the breast was observed, suggesting that the diet enhanced not only the amino acid digestibility, but also its absorption in the animals.

Furthermore, the increase of 4% (0.15 Mcal/kg) in energy digestibility observed in the current study is in concordance with the results reported by Peinado, et al. [27], who observed a similar magnitude, 3.8%, in the energy digestibility of broiler chickens diet supplemented with 90 mg of PTSO/kg and suggested that PTSO improved the intestinal structure. In addition, phosphorus digestibility showed a similar positive trend when 250 mg/kg of Pe-PTSO was added to the diet. Phosphorus is a non-renewable, expensive, and essential natural resource for agricultural production, so its digestibility must be assessed to reduce its excretion as much as possible. Excesses of N and P are associated with the eutrophication phenomenon that damages rivers, lakes, and oceans [45].

We suggest that improvement in the nutrient digestibility observed in our study could be due to better intestinal health, which has been reported by Peinado, et al. [26] and Ur Rahman, et al. [46], who supplemented broiler diets with *Allium* compounds, finding an increment in height and width of the intestinal villi, as well as a greater surface area, resulting in major absorption of nutrients. Moreover, it has been reported that PTSO modifies the gut microbiota, as the presence of enterobacteria decreased with this treatment, creating a better environment, reducing the negative effect of overgrowth of enterobacteria on the intestinal mucosa, and promoting the absorption of nutrients [27,47,48].

### 4.2. Productive Performance

Several studies have demonstrated the effects dietary supplementation with *Allium sativum* (garlic) and its secondary metabolites on productive performance and health in animals [22,23,49]. Kothari, et al. [50] supplemented poultry diets with *Allium* extracts; the results showed that the additive positively modulated bird growth, performance indices, lipid metabolism, and the gut ecosystem, as well as the immune response, especially under stressful and disease-challenged conditions. These findings indicate that garlic has a plethora of beneficial effects on the metabolism. Moreover, Brzóska, et al. [44] used a diet supplemented with a liquid garlic extract, and also reported improvements in the weights of broilers.

Not only have complete garlic extracts shown benefits, but also the secondary metabolites have been proven to impact growth performance [25,26]. In this sense, the results observed in our study demonstrate that dietary supplementation with 250 mg/kg of Pe-PTSO resulted in a significant increase in the ADG, compared to P0.

This result is in concordance with those presented by Kim, et al. [25], who offered a diet with 6.7 mg/kg of PTSO and 3.3 mg/kg of propyl-propane thiosulfinate (PTS) to broiler chickens challenged with *Eimeria acervulina* and reported that PTSO/PTS improved ADG and decreased fecal oocyst excretion compared with birds given a non-supplemented diet.

It is important to mention that in our study, the higher doses of Pe-PTSO, P500 and P750, did not show significant difference on ADG compared to P0, which may be explained because broiler chickens tended to decrease ADFI when the PTSO inclusion was increased from P250 to P500 or P750. Our results agree with Varmaghany, et al. [51], who observed that increasing the dose of garlic and its products reduces feed intake because it has a pungent smell and could reduce diet palatability [52]. Moreover, this effect is not exclusive for garlic and its products. Tahir, et al. [53] observed that using incremental doses of another phytochemical, eugenol, in broiler feed, affected the palatability, decreasing the ADFI while raising the inclusion of eugenol. Regarding to ION birds, our data suggests that it has no significant effect on performance.

Our results indicate that PTSO could enhance the performance of healthy broiler chickens when it is offered in a diet based on a corn–soybean meal. Moreover, Peinado, et al. [26] observed higher ADG when chicken diets were supplemented with 45 mg/kg of PTSO and a better FCR when chicken diets were supplemented with 45 or 90 mg/kg of PTSO. However, our findings did not show significant differences in the FCR. Nevertheless, it is worth mentioning that there are discrepancies between the various studies in which *Allium* derivatives have been used to improve production parameters in poultry nutrition [54,55,56]. These discrepancies may be caused by a number of reasons; attempting to explain this phenomenon, Ruiz, et al. [28] pointed out that the variation in the productive performance of broilers when fed products derived from garlic (*Allium sativum*) could arise because the chemically stable active compounds in these products were not characterized in all studies.

## 5. Conclusions

In summary, the inclusion of 250 mg of encapsulated PTSO per kg in the broiler chicken diet improved the digestibility of amino acids and energy, as well as the ADG. However, further research is needed to explain the mode of action and the correct dose of Pe-PTSO.

## Figures and Tables

**Table 1:** Ingredient and calculated chemical composition (g/kg as fed) and energy (Mcal/kg) of the basal diet.

Ingredient	g/kg
Yellow corn	513.9
Soybean meal	406.0
Vegetable oil	40.5
Calcium carbonate	14.7
Calcium orthophosphate	9.1
Sodium bicarbonate	4.9
Methionine DL	3.6
Refined salt	2.0
L-lysine HCl	2.2
L-Threonine	1.1
Betaine anhydrous	0.6
L-valine	0.2
Biocholine	0.2
Vitamins—mineral premix ^1^	0.9
Phytase 5000	0.1
**Chemical Composition**	**g/kg**
Dry matter	883.9
Crude protein	239.0
Crude fat	62.0
Gross energy (Mcal/kg)	4.0
Metabolizable energy (Mcal/kg)	3.15
Calcium	10.0
Total phosphorus	5.9
Available phosphorus	4.5
Sodium	2.3
Chloride	2.0
Potassium	9.5
DEB (mEq/kg) ^2^	300
Arginine	16.2
Lysine	15.0
Leucine	19.3
Threonine	10.2
Histidine	5.9
Isoleucine	10.1
Valine	11.2
Phenylalanine	11.8

^1^ Content per kilogram: vitamin A (retinol acetate), 12,000 International Units (IU); vitamin D3, 5000 IU; vitamin E (DL-α-tocopherol acetate), 50 IU; vitamin K, 3 mg; thiamine, 3 mg; riboflavin, 9 mg; pantothenic acid, 15 mg; pyridoxine, 4 mg; biotin, 0.2 mg; folic acid, 2 mg; vitamin B12, 0.02 mg; manganese, 100 mg; zinc 100 mg; iron, 40 mg; copper, 15 mg; iodine, 1 mg; selenium, 0.35 mg. ^2^ Dietary electrolyte balance (DEB).

**Table 2:** Apparent ileal digestibility of amino acids (%), energy (Mcal/kg), and phosphorus (%) in 20-day-old broiler chickens fed a corn–soybean diet supplemented with different inclusions of encapsulated propyl propane thiosulfonate (Pe-PTSO) or ION.

Treatment ^1^							
Nutrient ^2^	P0	P250	P500	P750	ION	SEM ^3^	*p* Value
Arg	94.47 ^b^	96.17 ^a^	95.04 ^ab^	94.46 ^b^	95.25 ^ab^	0.30	<0.01
Lys	94.03 ^b^	95.90 ^a^	95.01 ^ab^	94.40 ^b^	94.80 ^ab^	0.31	<0.01
Leu	92.22 ^b^	94.52 ^a^	93.31 ^ab^	92.75 ^ab^	93.34 ^ab^	0.42	0.01
Thr	90.25 ^b^	92.56 ^a^	91.39 ^ab^	90.57 ^b^	91.68 ^ab^	0.45	0.01
His	92.97 ^b^	95.17 ^a^	93.57 ^ab^	93.68 ^ab^	93.56 ^ab^	0.41	0.01
Ile	90.88 ^b^	93.92 ^a^	92.24 ^ab^	91.84 ^ab^	92.06 ^ab^	0.57	0.02
Val	90.29 ^b^	93.16 ^a^	91.56 ^ab^	91.27 ^ab^	91.28 ^ab^	0.55	0.02
Phe	92.52 ^b^	94.66 ^a^	93.58 ^ab^	92.95 ^ab^	93.45 ^ab^	0.47	0.05
Energy	3.39 ^b^	3.54 ^a^	3.47 ^ab^	3.40 ^b^	3.45 ^ab^	0.03	0.01
P	73.79	79.49	76.09	72.86	76.70	1.57	0.06

^a,b^ Different letters in the same row indicate significant differences (*p* ≤ 0.05). ^1^ P0, negative control, corn–soybean basal diet; P250, basal diet + 250 mg/kg Pe-PTSO; P500, basal diet + 500 mg/kg Pe-PTSO; P750, basal diet + 750 mg/kg Pe-PTSO; ION, positive control, basal diet + 50 mg/kg nicarbazin + 50 mg/kg narasin. ^2^ Arginine (Arg), lysine (Lys), leucine (Leu), threonine (Thr), histidine (His), isoleucine (Ile), valine (Val), phenylalanine (Phe), and phosphorus (P). ^3^ SEM, standard error of the mean, n = 5.

**Table 3:** Orthogonal polynomial contrasts trend analysis of the apparent ileal digestibility of amino acids, energy, and phosphorus in 20-day-old broiler chickens fed a corn–soybean diet supplemented with encapsulated propyl propane thiosulfonate (Pe-PTSO) ^1^.

	Linear Trend		Quadratic Trend		Cubic Trend	
Nutrient ^2^	*p* Value	R^2^	*p* Value	R^2^	*p* Value	R^2^
Arg	0.57	0.02	0.02	0.36	0.01	0.52
Lys	0.91	<0.01	0.01	0.40	0.01	0.52
Leu	0.88	<0.01	0.03	0.33	0.02	0.46
Thr	0.94	<0.01	0.03	0.35	0.02	0.45
His	0.84	<0.01	0.17	0.19	0.02	0.45
Ile	0.73	<0.01	0.06	0.28	0.02	0.45
Val	0.69	<0.01	0.03	0.26	0.02	0.44
Phe	0.94	<0.01	0.06	0.28	0.05	0.39
Energy	0.83	<0.01	0.02	0.37	0.02	0.47
P	0.49	0.03	0.04	0.31	0.05	0.37

^1^ P0, negative control, corn–soybean basal diet; P250, basal diet + 250 mg/kg Pe-PTSO; P500, basal diet + 500 mg/kg Pe-PTSO; P750, basal diet + 750 mg/kg Pe-PTSO. ^2^ Arginine (Arg), lysine (Lys), leucine (Leu), threonine (Thr), histidine (His), isoleucine (Ile), valine (Val), phenylalanine (Phe), and phosphorus (P).

**Table 4:** Growth performance of broiler chickens fed a corn–soybean diet supplemented with encapsulated propyl propane thiosulfonate (Pe-PTSO) or ION.

Treatments ^2^							
Parameters ^1^	P0	P250	P500	P750	ION	SEM ^3^	*p* Value
IBW (g)	284.2	282.9	286.9	289.2	289.6	2.18	0.16
ADG (g/d)	51.35 ^b^	57.33 ^a^	53.16 ^ab^	55.60 ^ab^	52.07 ^ab^	1.38	0.03
ADFI (g/d)	67.87	74.92	68.04	69.73	70.05	1.75	0.06
FCR (g/g)	1.32	1.31	1.28	1.26	1.34	0.03	0.23

^1^ IBW= initial body weight (10 d), ADG = average daily weight gain, ADFI = average daily feed intake, FCR = feed conversion ratio. ^2^ P0, negative control, corn–soybean basal diet; P250, basal diet + 250 mg/kg Pe-PTSO; P500, basal diet + 500 mg/kg Pe-PTSO; P750, basal diet + 750 mg/kg Pe-PTSO; ION, positive control, basal diet + 50 mg/kg nicarbazin + 50 mg/kg narasin. ^3^ SEM, standard error of the mean, n = 5. ^a,b^ Different letters in the same row indicate significant differences (*p* ≤ 0.05).

## Data Availability

The data presented in this study are available on request from the corresponding author.
